# Construction of an Interpretive Structural Model-Analytic Network Process model for preschool children’s drowning risk factors based on the Haddon matrix

**DOI:** 10.3389/fpubh.2026.1819414

**Published:** 2026-05-14

**Authors:** Feifei Ma, Dongmei Luo, Hongqiang Liu, Guohui Zhao

**Affiliations:** 1 School of Physical Education, Shanxi University, Shanxi, China; 2 School of Sport Science, Beijing Sport University, Beijing, China

**Keywords:** drowning, Haddon matrix, ISM-ANP model, preschool children, risk factors

## Abstract

**Objective:**

This study aims to systematically analyze the complex mechanisms of risk factors for drowning in preschool children, clarify their structural relationships and relative importance, and provide scientific evidence for constructing a precise and effective drowning prevention and control system for preschool children.

**Methods:**

Based on the theoretical framework of the Haddon matrix (which aims to systematically identify and categorize risk factors from the four dimensions of host (child), agent (water), physical environment, and social environment), the Interpretive Structural Model (ISM) and Analytic Network Process (ANP) were integrated. First, ISM was used to hierarchically deconstruct 18 risk factors, revealing their hierarchical transmission paths; then, ANP was utilized to quantify the global weights of each factor in the network and determine their relative impact levels.

**Results:**

(1) ISM analysis indicated that the risk factors presented a five-layer hierarchical structure: the surface layer included direct factors such as children’s risky behaviors (F4) and lack of immediate protection (F5); the middle layer centered around caregiver behaviors and supervision (F13); the bottom layer consisted of fundamental factors such as community safety systems (F15) and family economic background (F16). (2) Three key transmission chains were identified: social ecological pathways, direct pathways of the physical environment, and emergency response pathways. (3) ANP weight analysis showed that risky behaviors (28.06%) and caregiver supervision (23.26%) were the two core factors, with their combined weight exceeding 50%, followed by immediate response and lack of protection (14.69%).

**Conclusion:**

This study demonstrates that the drowning risk for preschool children stems from a complex, multi-factorial system transmission. Based on the principle of “structural layering and priority weighting” derived from our ISM-ANP model, we propose a three-tiered prevention and control strategy framework. This framework prioritizes short-term interventions targeting children’s risky behaviors and caregiver supervision, medium-term reinforcement of physical and environmental barriers, and long-term optimization of the socio-ecological foundation. The findings underscore that effective prevention requires a systematic governance approach. The proposed layered strategy provides a theoretical basis and decision-making reference for constructing a targeted and precise drowning prevention and control system for preschool children.

## Introduction

Drowning is one of the leading causes of injury-related death among children globally, posing a particularly prominent threat to preschool children. According to data from the World Health Organization (WHO), approximately 236,000 people die from drowning each year worldwide, with a high proportion being children and adolescents, and the drowning mortality rate in the 1–4 age group is the highest among all child age groups (1). In China, drowning is also the leading cause of injury-related death among children aged 1–14 (2). Preschool children have immature cognitive abilities, strong curiosity, and weak risk awareness, coupled with insufficient physical control and self-rescue capabilities, putting them at significant risk when exposed to water (3). Drowning not only leads to irreparable loss of life and heavy disease burdens but also brings profound economic and emotional impacts on families and society (4). Therefore, systematically identifying and accurately intervening in the risk factors for drowning in preschool children has become an urgent task in the fields of public health and child safety. Traditional drowning risk studies have often focused on single-factor analyses, such as swimming skills, caregiver supervision, or characteristics of water environments (5). However, drowning among children is essentially a complex system problem formed by the non-linear interactions of multi-dimensional factors, including individual, environmental, and social aspects (6). In recent years, systems thinking has gradually been introduced into the field of injury prevention, emphasizing the need to clarify the complex causal structures and interaction strengths among various risk factors to develop effective comprehensive intervention strategies (7).

To systematically deconstruct the complex risk factor system mentioned above, this study introduces an integrated analytical framework combining the Interpretive Structural Model (ISM) and the Analytic Network Process (ANP). Both are mature system modeling tools with strong analytical potential in the field of risk management. The Interpretive Structural Model (ISM) is a structured modeling technique based on graph theory. It identifies the direct and indirect logical relationships among elements in a complex system, transforming the intricate relationships among system elements into a clear, hierarchical multi-level structural model (8, 9). This model effectively reveals the hierarchy, transmission paths, and key nodes of risk factors and has been successfully applied in structural analysis of risk factors in fields such as building safety and traffic safety (10, 11). The Analytic Network Process (ANP) is an extension of the Analytic Hierarchy Process (AHP) for handling non-linear network structure problems. Unlike AHP, which assumes independence and strict hierarchical levels of elements, ANP allows for feedback and dependency relationships among elements, making it more aligned with the intertwined network state of risk factors in reality (12). ANP constructs a network structure that includes clusters of elements and their internal elements, calculating local and global weights through pairwise comparison judgment matrices to determine the relative importance ranking of each factor in the system (13). This characteristic makes it particularly suitable for addressing complex decision-making problems like drowning risk, where factors are interdependent and mutually influential.

By combining ISM and ANP, a complementary analytical framework can be formed: first, using ISM to perform topological sorting and hierarchical structuring of risk factors, clarifying their transmission mechanisms; then using the network structure output from ISM as input for ANP to calculate weights, thereby identifying the key risk factors and transmission paths that have the greatest impact on the system. This ISM-ANP integrated model has been applied in evaluations of complex systems such as energy security and supply chain resilience (14, 15), but its application in the field of child injury prevention, especially in drowning risk analysis, is still in the exploratory stage, providing methodological innovation space for this study.

To construct a comprehensive and structured list of risk factors, which will serve as the analytical basis for the ISM-ANP model, it is necessary to rely on a systematic and mature theoretical framework for guidance. The Haddon matrix, as a classic analytical tool in the field of injury prevention, provides appropriate theoretical support for this purpose. This matrix was proposed by Dr. William Haddon Jr., a pioneer in injury epidemiology, and is based on three dimensions: host (individual or population), agent/vehicle (such as water bodies as energy transfer vehicles), and physical/social environment, combined with three time phases: pre-event, event, and post-event, forming a 3 × 3 structured analytical grid (16). Its core value lies in guiding researchers to systematically identify the risk factors, protective mechanisms, and response strategies involved in each dimension throughout the entire process of injury occurrence, development, and outcome (17, 18).

In drowning prevention research, the Haddon matrix has been widely used as a core analytical framework, providing a structured perspective for systematically sorting and categorizing intervention measures. For example, in the pre-event phase, corresponding intervention strategies can be categorized into different dimensions: the host dimension mainly includes conducting water safety education for children; the agent/environment dimension encompasses setting up pool fences (engineering interventions) and installing water depth warning signs; the social environment dimension involves formulating and enforcing regulations prohibiting swimming in areas without lifeguard supervision (enforcement interventions) (19–21). However, traditional Haddon matrices mainly focus on the static classification and listing of factors, lacking in-depth quantitative analysis of the interaction relationships, hierarchical structures, and comprehensive contributions of various factors to drowning risk (22). This limitation somewhat weakens its practical effectiveness in guiding the formulation of precise and prioritized intervention strategies. Therefore, combining the systematic classification advantages of the Haddon matrix with the structural and weight analysis capabilities of the ISM-ANP model to construct a composite analytical model that can both identify risk networks and quantify their internal mechanisms has become an important direction for enhancing the scientific and practical guiding value of drowning risk research in preschool children.

This study integrates the Haddon matrix, the Interpretive Structural Model (ISM), and the Analytic Network Process (ANP) to construct a systematic analysis model for risk factors of drowning in preschool children. The study aims to achieve the following objectives: (1) Based on literature research and expert consultation, use the Haddon matrix to systematically identify and classify key risk factors; (2) Clarify the logical relationships among factors using the ISM method and establish a hierarchical Interpretive Structural Model; (3) Based on the structure constructed by ISM, use ANP to quantify the mutual influences among factors, calculate their comprehensive weights, and identify key risk factors and main transmission paths. By clarifying the structural relationships and relative importance among risk factors, this study expects to provide a basis for optimizing the allocation of limited prevention resources, enhancing the targeting and cost-effectiveness of intervention measures, and promoting the scientific and precise development of drowning prevention strategies for preschool children. This study proposes the following core hypotheses: the risk factors for drowning in preschool children constitute a complex network system; the factor network categorized based on the Haddon matrix and structured by ISM can undergo effective weight analysis through ANP; the analysis results can reveal key risk nodes and transmission mechanisms that are difficult to identify through traditional methods, thereby providing scientific evidence for precise interventions that go beyond empirical judgments.

## Methods

### Preliminary screening of influencing factors and system construction

To systematically identify the risk factors for drowning in preschool children, this study constructed an initial pool of risk factors by combining literature analysis and expert consultation methods. First, a systematic search was conducted for Chinese and English literature on children’s drowning risks and prevention from the initial date to 2025 in databases such as CNKI, Web of Science, and PubMed, and relevant guidelines and reports published by the World Health Organization (WHO) and the Chinese Center for Disease Control and Prevention (CDC) were referenced (1, 23, 24). Based on the Haddon matrix framework (host, agent/vehicle, physical and social environment × pre-event, event, post-event), a preliminary extraction and classification of risk factors were performed, forming an initial list of factors covering multiple dimensions such as cognition, behavior, environment, supervision, and emergency response.

To enhance the comprehensiveness and practical relevance of the factors, a Delphi method was further employed for two rounds of expert consultation. The consultation group consisted of 8 experts, including injury epidemiology researchers (2), pediatricians (2), emergency responders (2), and community safety managers (2). In the first round of consultation, experts reviewed the preliminary list, supplemented, merged, deleted, and revised items, and assessed the importance of each factor in the context of preschool children’s drowning situations using a 5-point Likert scale. After integrating and revising the factors based on the feedback from the first round, a second round of consultation was conducted for experts to confirm the revised list. Ultimately, a total of 50 core risk factors were identified based on the criteria of an average importance score ≥4.0 and a coefficient of variation <0.25 (Table 1), establishing the initial risk factor system for this study and providing a foundation for subsequent questionnaire development.

**Table 1:** Risk factors for drowning in preschool children Haddon matrix.

**Stage**	**Host**	**Medium**	**Physical environment**	**Social environment**
	**Preschool children**	**Water areas and equipment**		
Before drowning	Age, gender, cognitive level, underlying diseases, lack of swimming survival skills, lack of strength, hyperactivity or impulsive personality, overestimation of one’s swimming skills	Equipment without protective water hazards, unsafe vessels or overcrowding, substandard water recreation facilities, water temperature too low, lack of personal flotation devices or other lifesaving equipment on board	Lack of barrier protection around water areas, slippery, uneven, or steep surfaces near water, seasons like summer, lack of depth markings or warning signs in pools and other water areas, uncovered household water storage containers, high exposure of water sources inside or around the home, lack of escape mechanisms like ropes, steep and slippery banks difficult to climb	Lack of supervision for young children, identity of young children (urban or rural), caregivers lacking first aid knowledge, large family size, authorities failing to address risk factors, caregivers engaging in behaviors like drinking or distraction, reliance on peers or older children for supervision, caregivers overestimating children’s swimming skills, caregivers’ insufficient awareness of drowning risks, lack of safety patrol personnel around water areas, lack of guidance on water safety and community outreach programs, low educational level of caregivers, low-income families, schools not conducting systematic water safety education, lack of established drowning emergency response mechanisms
During drowning	Fear response in young children, young children not wearing personal flotation devices, young children bathing or swimming alone	Water depth, victim being swept away by currents	No lifeguard	Bystanders not reporting or calling for help in time
After drowning				Difficult transportation for providing medical assistance, inadequate communication for calling emergency medical services, incorrect rescue and handling techniques, difficulty accessing emergency care hospitals and rehabilitation services, delayed response time from emergency or fire departments, lack of advanced medical equipment and technology, families unable to afford subsequent rehabilitation costs

### Questionnaire design and reliability and validity testing

#### Questionnaire compilation and data collection

Based on the 50 risk factors selected in the previous stage, this study compiled the “Importance Evaluation Questionnaire for Risk Factors of Drowning in Preschool Children.” The questionnaire employed a 5-point Likert scoring method, inviting experts and relevant practitioners in the field of child safety (such as kindergarten managers and community health workers) to evaluate the potential contribution of each factor to drowning incidents among preschool children (1 = very unimportant, 5 = very important). A total of 220 questionnaires were distributed through a professional online survey platform, with 205 valid responses collected, resulting in an effective response rate of 93.2% (see Table 2). The collected data will be used for subsequent analyses using the Interpretive Structural Model (ISM) and the Analytic Network Process (ANP). All participants were informed and provided informed consent. This study was approved by the Ethics Committee of Shanxi University Medical (Approval No.: SXULL2024116).

**Table 2:** Basic characteristics of survey subjects.

Parameters	Gender		Professional background				Work experience
	Male	Female	Researchers	Pediatricians	Emergency rescue	Kindergarten workers	≥3 years
Number	87	118	55	48	52	50	205
Proportion (%)	42.4	57.6	26.8	23.4	25.4	24.4	100

#### Reliability and validity analysis of the questionnaire

To ensure the scientific nature of the research tool and the reliability of the measurement results, reliability and validity tests were conducted on the questionnaire data.

Reliability testing: The internal consistency reliability index Cronbach’s *α* coefficient was used to evaluate the overall questionnaire and each dimension of the Haddon matrix. The results showed that the overall Cronbach’s *α* coefficient of the questionnaire was 0.937, and the α coefficients for each dimension (host, agent, physical environment, social environment; pre-event, event, post-event) were all above 0.85, indicating excellent internal consistency and measurement reliability of the questionnaire.

Validity testing: This mainly included content validity and structural validity. Content Validity: The questionnaire items were based on a systematic literature review and underwent two rounds of expert consultation for selection and revision, ensuring the representativeness and relevance of the content. Structural validity: Exploratory factor analysis (EFA) was used for testing. First, the KMO (Kaiser-Meyer-Olkin) sampling adequacy measure and Bartlett’s test of sphericity were used to assess whether the data were suitable for factor analysis. The results showed that the KMO value was 0.912 (>0.8), and the approximate *χ*^2^ value of Bartlett’s test was 12568.34 (*p* < 0.001), indicating that the data were very suitable for factor analysis. Subsequently, principal component analysis was used to extract factors, with an eigenvalue >1 as the criterion, and orthogonal rotation using the maximum variance method to clarify the factor structure. Ultimately, 18 common factors were extracted from the 50 risk factors (see Table 3), which could explain most of the information of the original variables well, indicating that the questionnaire has good structural validity. The 18 extracted common factors will serve as the basic analytical elements for constructing the ISM-ANP model in subsequent analyses.

**Table 3:** Variance contribution rates of initial eigenvalues of factors.

Dimensions	Code	Eigenvalue	Variance contribution rate/%	Cumulative contribution rate/%
Preschool children	F1	3.855	35.047	35.047
	F2	2.105	19.135	54.182
	F3	1.411	12.826	67.008
	F4	1.274	11.583	78.591
	F5	1.073	9.758	88.349
Water area and equipment	F6	3.381	42.256	42.256
	F7	2.376	29.695	71.952
	F8	1.244	15.556	87.508
Physical environment	F9	3.722	41.355	41.355
	F10	1.573	17.48	58.836
	F11	1.478	16.424	75.26
	F12	1.039	11.55	86.81
Social environment	F13	4.569	24.05	24.05
	F14	4.188	22.04	46.09
	F15	2.323	12.225	58.315
	F16	2.195	11.552	69.867
	F17	2.039	10.732	80.599
	F18	1.134	5.966	86.566

### Principles and steps for constructing the ISM-ANP composite model

To systematically analyze the structural relationships and relative importance of risk factors for drowning in preschool children, this study integrates the Interpretive Structural Model (ISM) and the Analytic Network Process (ANP) to construct a two-phase composite model of “structural analysis-weight calculation.” ISM is used to qualitatively reveal the hierarchical structure and transmission paths among common factors, while ANP performs quantitative weight calculations based on the hierarchical structural relationships revealed by ISM.

#### Construction of the Interpretive Structural Model (ISM)

The Interpretive Structural Model aims to analyze the structural relationships among elements in a complex system. This study uses ISM to logically organize the 18 common factors (F1–F18) extracted from exploratory factor analysis and transform them into a structured model with multi-level hierarchical characteristics. The specific construction steps are as follows:

(1) Determining system elements: The 18 common factors obtained from factor analysis are identified as the system elements for ISM analysis. (2) Constructing the adjacency matrix: A small group of 5 domain experts is formed to judge whether there is a direct causal relationship between any two common factors based on theoretical literature and empirical evidence, thereby constructing an 18 × 18 binary adjacency matrix A. (3) Calculating the reachability matrix: The system reachability matrix M is solved using Boolean algebraic operations. This matrix represents the indirect reachability relationships among elements after any path length, with the calculation formula M=(A+I)^k,iterating until(A+I)^k=(A+I)^{k+1} is satisfied, where I is the identity matrix. This step is implemented through MATLAB R2023a software programming. (4) Hierarchical division and model visualization: Based on the reachability matrix M, hierarchical division is performed by analyzing the antecedent set, reachable set, and intersection set of each element. Finally, the elements with hierarchical relationships and their directed connections are visually presented, drawing a multi-level hierarchical structure model diagram of risk factors for drowning in preschool children, thereby intuitively revealing the transmission paths of risks from deep-rooted causes to surface manifestations.

#### Weight calculation based on the Analytic Network Process (ANP)

The Analytic Network Process is suitable for handling complex decision-making problems where there are interdependencies and feedback relationships among system elements. This study quantifies the relative importance of each risk factor in the system using ANP based on the hierarchical structural relationships output by the ISM model. The main steps are as follows:

(1) The 18 common factors are defined as “elements” in the ANP network, and based on the theoretical framework of the Haddon matrix and the influence relationships revealed by ISM, they are categorized into four element sets: “host factors,” “agent factors,” “physical environment factors,” and “social environment factors” (i.e., network clusters). The direct and indirect influence relationships identified by ISM are converted into dependency and feedback paths among elements within and between clusters in the ANP network, thereby constructing a complete non-linear network structure model. (2) Constructing judgment matrices and calculating local weights: Using expert evaluation methods and Saaty’s 1–9 scale, pairwise comparisons of the importance of each element are conducted for the upper criteria affected by common influences, constructing a series of judgment matrices. All judgment data are processed by yaanp 2.0 software, which calculates the local weight vectors of each judgment matrix based on the eigenvalue method and performs consistency checks (requiring consistency ratio CR < 0.1). (3) Integrating super matrices and calculating comprehensive weights: The software integrates all local weights into an unweighted super matrix. Subsequently, based on the relative importance of each element set under the control layer objective (obtained through pairwise comparisons), it is transformed into a weighted super matrix. Finally, by calculating the limit of this weighted super matrix (i.e., finding its power limit), a stable limit super matrix is obtained, where any column vector represents the global comprehensive weight of each risk factor. This weight fully considers the interdependencies and feedback relationships within the system, reflecting the relative importance of each factor in the entire risk network. (4) Through the above steps, this study completes the complete analytical process from the structural analysis of risk factors to the quantification of their system importance, laying a methodological foundation for identifying key risk nodes, paths, and proposing precise intervention strategies in subsequent analyses.

## Results

### Basic characteristics of survey subjects

A total of 205 valid questionnaires were collected in this study. The survey subjects mainly came from fields related to child health, safety education, and public health, including 87 males (42.4%) and 118 females (57.6%). In terms of professional background, there were 55 injury epidemiology and prevention researchers (26.8%), 48 pediatric clinical doctors and nurses (23.4%), 52 public health institution managers (25.4%), and 50 kindergarten and community safety education workers (24.4%). All participants had more than 3 years of relevant work experience, ensuring the professionalism and reliability of the questionnaire assessment, providing a solid data foundation for the accurate identification of risk factors in subsequent analyses.

### Establishment of the ISM model for risk factors of drowning in preschool children

#### Extraction and naming of common factors

To reduce dimensionality and reveal the internal structure of the questionnaire data, this study employed factor analysis. First, KMO tests were conducted on the three dimensions involved in the questionnaire, with values of 0.82, 0.79, and 0.84, all >0.50, indicating good correlations among variables and suitability for factor analysis. Subsequently, dimensionality reduction was performed on the 50 specific influencing factors, extracting common factors based on an eigenvalue >1 and rotating using the maximum variance method, requiring a cumulative variance contribution rate of 85% (25). Ultimately, 18 common factors were extracted (see Table 3).

Factor analysis aims to aggregate highly correlated observed variables into the same latent construct (i.e., common factors), thereby revealing the internal structure among variables. After extracting common factors, they need to be professionally named based on their high-loading variables and theoretical meanings. For example, the variance contribution rate of common factor F1 in the “host” dimension is 35.047%, with high-loading variables including the age, gender, and urban–rural identity of preschool children, all of which belong to the category of individual attributes in the Haddon matrix (Table 1), thus naming F1 as “inherent characteristics of individuals.” The remaining common factors were named following this principle, with specific results shown in Table 4. The final 18 common factors cover the three dimensions of the Haddon matrix, with their cumulative variance contribution rates exceeding 85% (Table 3), indicating that these common factors can retain the information structure of the original 50 influencing factors well, demonstrating good explanatory power and structural validity, making them suitable as the basic analytical units for constructing the ISM-ANP model in subsequent analyses.

**Table 4:** Risk factors for drowning in preschool children.

**Preschool children**		**Water areas and equipment**		**Physical environment**		**Social environment**	
Code	Risk factors	Code	Risk factors	Code	Risk factors	Code	Risk factors
F1	Individual inherent characteristics	F6	Safety defects in equipment and facilities	F9	Lack of protective facilities in places	F13	Caregiver behavior and supervision
F2	Physical and mental abilities and health status	F7	Risks related to vessels	F10	Environmental risks	F14	Caregiver knowledge and cognition
F3	Water skills and cognitive biases	F8	Characteristics of water environment	F11	Family environmental exposure	F15	Community safety and education system
F4	Risky behaviors			F12	Supervision and time–space factors	F16	Family economic background
F5	Lack of immediate response and protection					F17	Emergency preparedness and response mechanisms
						F18	Medical rescue and follow-up support

#### Determination of binary relationships among common factors

To determine the direct causal relationships among common factors and construct the adjacency matrix for ISM, this study formed a consultation group consisting of 5 experts in drowning prevention and injury prevention. Using the Delphi method, systematic pairwise relationship determinations were conducted for the 18 common factors through multiple rounds of deliberation. After several iterations, the experts reached a high consensus (consensus rate >80%), ultimately clarifying the direct influence logic among common factors. The binary relationship between common factors F_i_ and F_j_ is defined as follows:


$$F_{\mathrm{ij}}=\{\begin{array}{c} V\;\text{factor}\;i\;\text{affects factor}\;j\\ A\;\text{factor}\;j\;\text{affects factor}\;i\\ X\;\text{factor}\;i\;\text{and factor}\;j\;\text{influence each other}\\ O\;\text{factor}\;i\;\text{and factor}\;j\;\mathrm{do}\;\text{not affect each other} \end{array}$$

**Figure 1:**
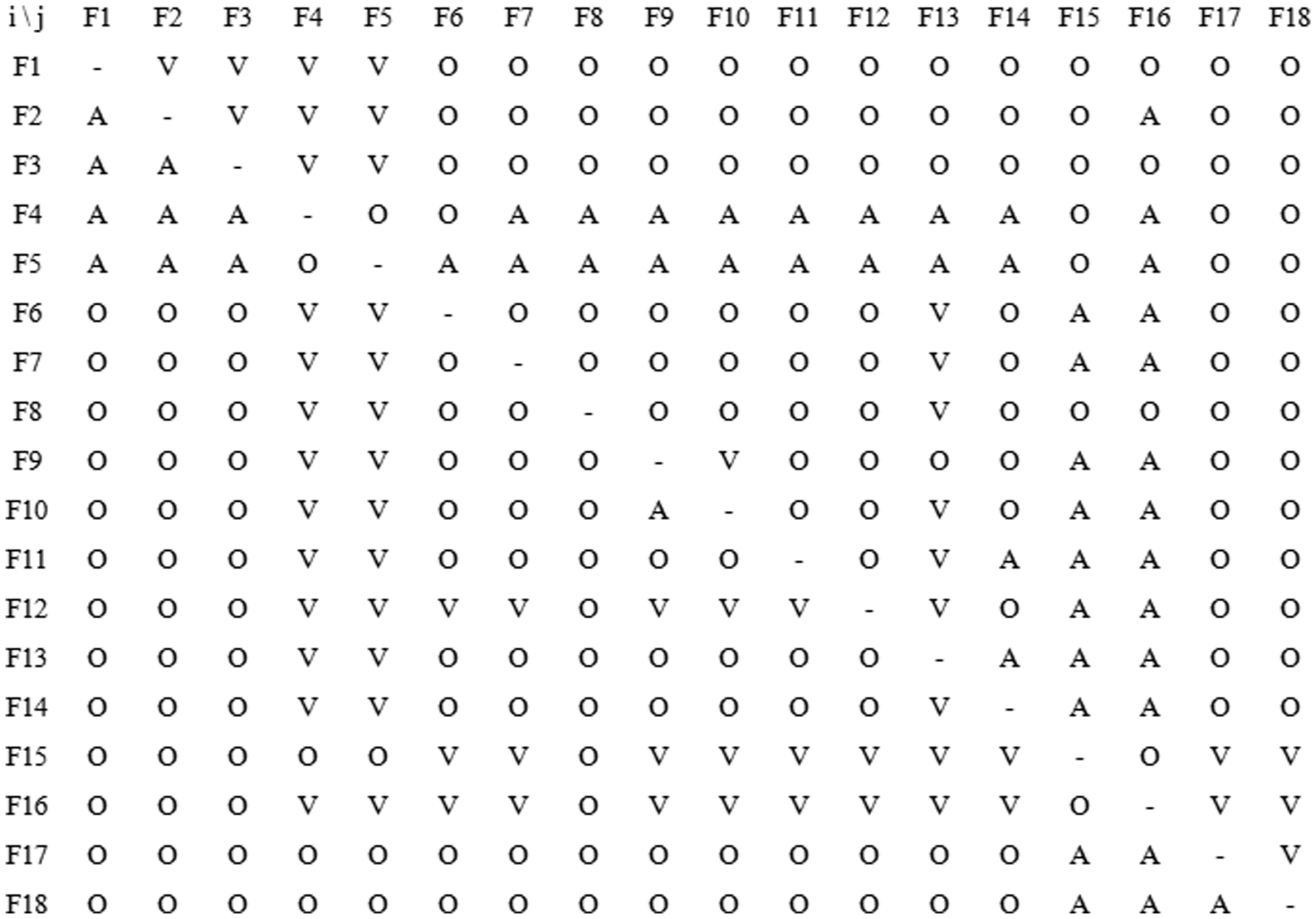
Binary logistic relationship of drowning risk factors in preschool children.

The binary logical relationships among factors are shown in Figure 1.

#### Construction of the adjacency matrix

**Figure 2:**
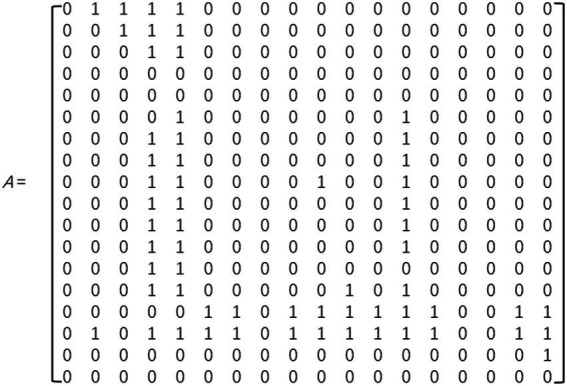
Adjacency matrix A.

To formally represent the direct influence relationships among common factors, this study constructed an adjacency matrix (Adjacency Matrix) A. The assignment rules for the matrix elements aij are as follows: (1) When i = j, a_ij_ = 0; (2) When i ≠ j, if the relationship is V, meaning F_i_ directly influences F_j_, then a_ij_ = 1, a_ji_ = 0; if the relationship is A, meaning F_j_ directly influences F_i_, then a_ij_ = 0, a_ji_ = 1; if the relationship is X, meaning F_i_ and F_j_ mutually influence each other, then a_ij_ = aj_i_ = 1; if the relationship is O, meaning there is no direct influence between F_i_ and F_j_, then a_ij_ = a_ji_ = 0. After assigning values according to the above rules, the resulting adjacency matrix A is shown in Figure 2.

#### Calculation of the reachability matrix

**Figure 3:**
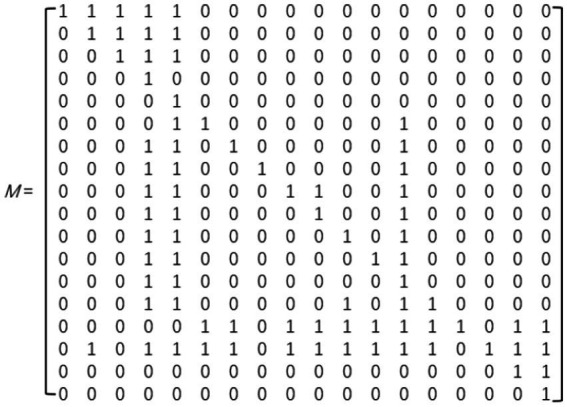
Reachable matrix M.

The Reachability Matrix (M) is used to represent whether there are direct or indirect associative paths between any two elements in the system and is a transitive extension of the relationships in the adjacency matrix. Its calculation is based on Boolean algebraic operations, using the adjacency matrix A and the identity matrix I (26), solving through matrix exponentiation iteration. Specifically, the power of the matrix (A + I) is calculated until it satisfies (A + I)^k = (A + I)^{k + 1}, at which point the matrix sequence converges to stability, and the resulting M = (A + I)^k is the reachability matrix of the system (Figure 3). This matrix fully expresses all reachability relationships among risk factors. The above iterative calculation process is implemented through MATLAB R2023a software programming, ensuring accuracy and efficiency in the calculations.

#### Hierarchical structure division of influencing factors

To reveal the hierarchical positions of common factors in the system structure, this study performed systematic hierarchical division calculations based on the reachability matrix M using MATLAB R2023a software, ultimately determining the hierarchical structural relationships of influencing factors (results shown in Table 5). The specific steps include calculating the reachable set R(Fi) (the set of factors reachable from Fi), the antecedent set Q(Fi) (the set of factors that can reach Fi), and their intersection R(Fi)∩Q(Fi). These set operations accurately represent the structural dependencies and hierarchical relationships among factors, with detailed data shown in Table 6.

**Table 5:** Risk factors for drowning in preschool children: ISM model hierarchical decomposition.

Hierarchy L	Common factor Fi
1	4, 5, 18
2	3, 13, 17
3	2, 6, 7, 10, 11, 12
4	1, 9, 14
5	15, 16

**Table 6:** Reachable set, antecedent set and their intersection.

F_i_	Reachable set R(Fi)	Antecedent set Q(Fi)	R(F_i_)∩Q(F_i_)
1	1–5	1	1
2	2–5	1, 2	2
3	3–5	1–3	3
4	4	1–4, 7–14, 16	4
5	5	1–3, 5–14, 16	5
6	5, 6, 13	6, 15, 16	6
7	4, 5, 7, 13	7, 15, 16	7
8	4, 5, 8, 13	8	8
9	4, 5, 9, 10, 13	9, 15, 16	9
10	4, 5, 10, 13	9, 10, 15, 16	10
11	4, 5, 11, 13	11, 14–16	11
12	4, 5, 12, 13	12, 15, 16	12
13	4, 5, 13	6–16	13
14	4, 5, 11, 13, 14	14–16	14
15	6, 7, 9–15, 17, 18	15	15
16	2, 4–7, 9–14, 16–18	16	16
17	17, 18	15–17	17
18	18	15–18	18

#### Drawing the ISM model diagram

**Figure 4:**
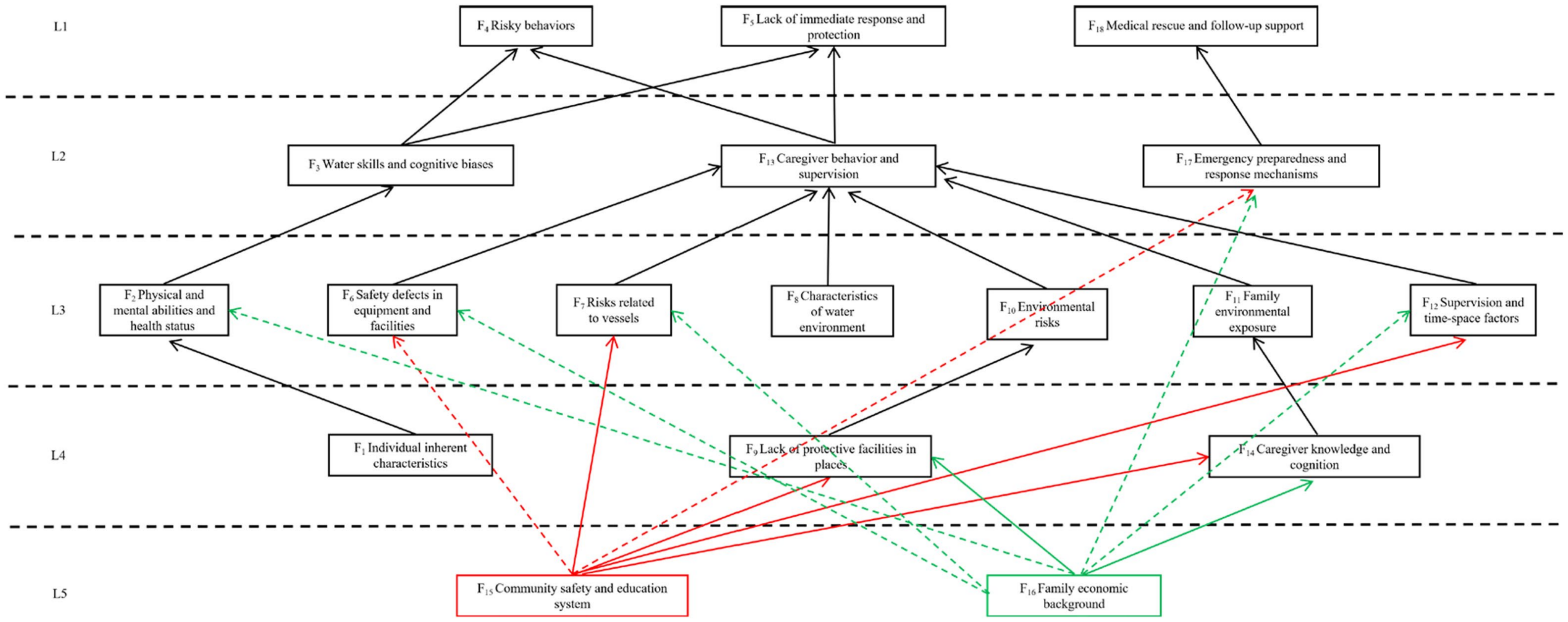
Risk factors for drowning in preschool children ISM model.

Based on the aforementioned hierarchical division results and the reachability relationships among common factors, this study drew a multi-level hierarchical Interpretive Structural Model of risk factors for drowning in preschool children, as shown in Figure 4. To clearly express the path relationships, F15 and F16 are represented by arrows of different colors. This model decomposes the 18 risk common factors into 5 clear levels (L1 to L5), forming a typical multi-level hierarchical structure. In the model, the factors are connected by directed arrows, with the arrow direction indicating the direction of causal relationships. The overall structure indicates that the lower-level factors are the driving sources or deeper causes of the upper-level factors, with risks transmitted stepwise along the arrow direction from deep-rooted causes (such as L5, L4) to surface manifestations (such as L2, L1). This structural model not only visually presents the hierarchical architecture of risk factors but also systematically reveals the complete transmission path of drowning risk from macro social environments and physical environments to individual behaviors and characteristics, providing a clear structured view for understanding the systemic mechanisms of risk occurrence.

### Weight calculation of factors based on ANP

#### Constructing the network structure

Based on the principles of the Analytic Network Process (ANP), this study divided the risk factor system for drowning in preschool children into control and network layers. The control layer includes the overall evaluation objective, namely “system analysis of risk factors for drowning in preschool children.” The network layer consists of interrelated and feedback elements, with its structure integrating the theoretical dimensions of the Haddon matrix and the structural relationships revealed by the aforementioned ISM model.

**Figure 5:**
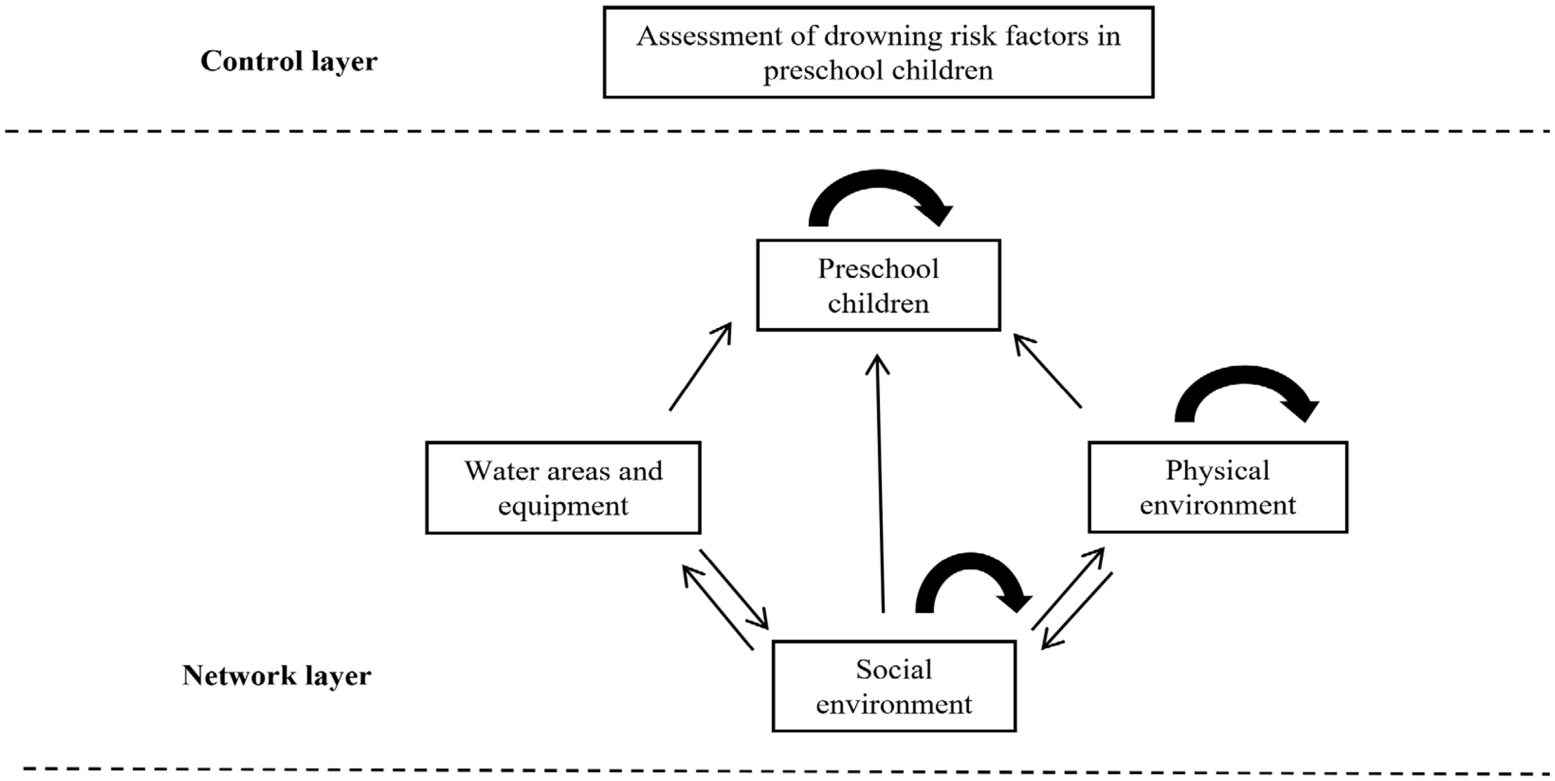
Network structure diagram of assessment of drowning risk factors in preschool children.

Specifically, the 18 common factors output by the ISM model are identified as the basic “elements” in the ANP network. According to the four categories of the Haddon matrix—host factors, agent factors, physical environment factors, and social environment factors—these elements are categorized into their respective four “element sets” (i.e., network clusters). The dependency and feedback relationships within each element set and among different element sets are constructed based on the direct and indirect influence paths identified by the ISM model, thereby forming a non-linear network structure model that aligns with the complexities of reality. The network structure model of the evaluation indicators is shown in Figure 5, providing a framework for subsequent pairwise comparisons and weight calculations.

#### Constructing the unweighted super matrix

Using Saaty’s 1–9 scale, pairwise comparisons of the importance among indicators were conducted to form the initial judgment matrix P.


$$P=[\begin{array}{ccc} p_{1,1} & \ldots & p_{1,n}\\ \vdots & \; & \vdots \\ p_{n,1} & \ldots & p_{n,n} \end{array}]$$

Subsequently, normalization processing was performed on all judgment matrices based on the eigenvalue method, and consistency checks were implemented to obtain the unweighted super matrix $W_{0}$ .


$$W_{0}=[\begin{array}{ccc} W_{1,1} & \ldots & W_{1,n}\\ \vdots & \; & \vdots \\ W_{n,1} & \ldots & W_{n,n} \end{array}]$$

#### Calculating the weighted super matrix

Using the same pairwise comparison method, a relative weight matrix A for the first-level indicators was established, and then the unweighted super matrix $W_{0}$ was subjected to Hadamard product operations with A to obtain the weighted super matrix W.


$$W=A\;o\;W_{0}=[\begin{array}{ccc} a_{1,1}W_{1,1} & \ldots & a_{1,n}W_{1,n}\\ \vdots & \; & \vdots \\ a_{n,1}W_{n,1} & \ldots & a_{n,n}W_{n,n} \end{array}]$$

#### Calculating the limit super matrix

The power method was used to iteratively calculate the powers of the weighted super matrix W until the matrix converged to a stable and unique state, ultimately obtaining the limit super matrix W∞, which serves as the basis for the global weights of each factor.


$$W_{\infty }=\lim_{k\to \infty }W^{k}$$

#### Calculating indicator weights

Based on the results of the limit super matrix W∞, the weights of the indicators for risk factors of drowning in preschool children were derived, denoted as vector F = [f₁, f₂, …, fₙ], with specific values shown in Table 7.

**Table 7:** Weight of risk factors indicators for drowning in preschool children.

Factor	Name	Global weight	Ranking
F4	Risky behaviors	0.2806	1
F13	Caregiver behavior and supervision	0.2326	2
F5	Lack of immediate response and protection	0.1469	3
F3	Water skills and cognitive biases	0.0709	4
F2	Physical and mental abilities and health status	0.069	5
F14	Caregiver knowledge and cognition	0.0405	6
F6	Safety defects in equipment and facilities	0.0355	7
F18	Medical rescue and follow-up support	0.0329	8
F9	Lack of protective facilities in places	0.0326	9
F11	Family environmental exposure	0.0231	10
F17	Emergency preparedness and response mechanisms	0.014	11
F10	Environmental risks	0.0111	12
F12	Supervision and time–space factors	0.0072	13
F7	Risks related to vessels	0.0031	14
F1	Individual inherent characteristics	0	15
F8	Characteristics of water environment	0	15
F15	Community safety and education system	0	15
F16	Family economic background	0	15

## Discussion

This study systematically analyzed the structural relationships and relative importance of 18 drowning risk factors for preschool children through the integration of Interpretive Structural Modeling (ISM) and Analytic Network Process (ANP). The analysis results not only revealed the hierarchical structure and transmission mechanisms of risk factors but also quantified the global influence of each factor within the system, thereby providing a solid scientific basis for constructing precise prevention and control strategies that are hierarchical and focused.

### ISM model analysis: hierarchical deconstruction and transmission mechanism of the risk system

The ISM model clearly reveals the dominance and dependence relationships among risk factors through the operations of reachable sets (R(Fi)), antecedent sets (Q(Fi)), and their intersections, thus dividing them into five hierarchical levels and depicting the dynamic transmission path of risk from deep-rooted causes to surface events.

#### Hierarchical structure explanation

Level L1 (surface direct factors: F4, F5, F18) constitutes the immediate proximal causes and consequences of drowning events. F4 (risky behavior) and F5 (lack of immediate response and protection) are the final manifestations of risk transmission to individual behavior, consistent with safety science theories such as Reason’s “Swiss cheese” model (27). For example, the lack of adult supervision for children playing in water (F4) and not wearing life jackets (F5) have been empirically confirmed as direct key situations leading to drowning (28, 29). F18 directly determines the severity of injury consequences, emphasizing the critical importance of rescue and medical support in the “drowning survival chain” (30, 31). Recent studies further point out that these direct factors do not exist in isolation: children’s impulsivity (part of F4) often co-varies with lower-level family environmental factors (32); panic responses (part of F5) can accelerate physical exhaustion, thereby exacerbating severity (33). This indicates that there are dynamic interactions within L1 factors, which may act as feedback nodes influencing system cycles. Structurally, the large antecedent set and smaller reachable set of L1 indicate that it is a “resulting” indicator of the combined effects of many lower-level factors.

Levels L2 and L3 (intermediate driving factors: F2, F3, F6, F7, F10, F11, F12, F13, F17) constitute the hubs and bridges of risk transmission. They are both products of deep social and environmental factors and proximate causes that trigger surface direct behaviors. Among them, F13 (caregiver behavior and supervision) is particularly critical: its reachable set R(F13) = {4,5,13} indicates it directly drives F4 and F5; while its antecedent set Q(F13) = {6–16} is extensive, making it a core intermediary node for risk aggregation and amplification. Research has confirmed that caregiver distraction or brief absence is one of the most critical proximate environmental factors, directly driving risky behavior and lack of protection (5, 28, 34). Other intermediate factors covered by the model (such as family environmental exposure, children’s water skills, etc.) have also been independently identified as important risk predictors (1), collectively supporting the model’s definition of the intermediate level as the “risk transmission hub.” Additionally, there may be feedback loops between intermediate and surface factors, for example, children’s impulsive traits (F2) may challenge supervisory patience (F13), thereby exacerbating risky behavior (F4), forming a dynamic vicious cycle (35, 36).

Levels L4 and L5 (deep-rooted factors: F1, F9, F14, F15, F16) are located at the bottom of the model and are the fundamental, foundational causes that trigger the entire risk system. F15 (community safety and education system) and F16 (family economic background) have extensive reachable sets, and their influence can transcend multiple levels to ultimately affect behavior and consequences. A large body of evidence indicates that family socioeconomic status (F16) is one of the strongest fundamental factors predicting drowning risk in children, acting through multiple pathways such as limiting access to safety resources, affecting living environments, and compressing caregiving energy (37, 38). Community-level interventions (F15), such as installing protective barriers (F9) and conducting public education, have been recognized by organizations like the World Health Organization as “the most effective strategies” because they can systematically benefit the entire population (23, 39, 40). At the same time, interventions targeting caregivers’ risk perception and responsibility awareness (F14) are more fundamental and lasting than simple first aid knowledge training (41). There are strong interactions and co-variation relationships among these deep factors, for example, low family income (F16) is often associated with a lack of community educational resources (F15) and high-risk living environments (F9), forming a “risk aggregation” effect (38, 42). Additionally, the risk effects of inherent individual characteristics (such as gender, F1) may also be moderated by social gender roles rather than purely biological attributes (43, 44), further reflecting the complexity of deep factors’ effects.

#### Key risk transmission path analysis

Based on a systematic analysis of the reachable matrix, this study further identified three dominant risk transmission paths for drowning in preschool children, which collectively reveal the core mechanisms of risk transmission from macro systems to micro events.

Social ecological path: F15/F16 (community/family level) → F13/F14 (caregiver level) → F4/F5 (children’s behavior level). This path is the most core transmission chain, highlighting the inherent logic of how the macro social environment profoundly influences children’s safety behaviors by shaping the micro family and caregiver environment. This path aligns closely with the classic “social ecological model” (45) and is entirely consistent with the multi-level intervention strategies advocated by the World Health Organization (WHO) from policy, community, interpersonal to individual levels (23). Recent studies have refined its transmission mechanism: for example, low socioeconomic status (F16) not only limits families’ access to safety resources but also damages the quality of effective supervision (F13) through increasing parental stress and reducing parent–child interaction time via psychosocial mechanisms, thereby directly increasing children’s risky behavior (F4) (46, 47). This empirically supports the core logic of macro factors transmitting through the caregiver level.

Physical environment direct path: F9/F10/F11/F12 (environment and supervision) → F4/F5 (children’s behavior level). This path indicates that unsafe physical environments and lack of supervision can directly induce risky behavior or lead to protection failures. Research shows that eliminating or isolating environmental hazards through engineering means (such as installing protective barriers, F9) is one of the most effective strategies for preventing drowning in children (39), as it can physically block children’s access to dangerous water areas, fundamentally preventing the occurrence of F4-type behaviors. Similarly, effective supervision in open water areas (such as lifeguards, F12) can directly stop risky behavior and respond immediately to incidents, which is a key link in reducing F5 (lack of immediate response) (48). Therefore, improving the physical environment and strengthening direct supervision is a direct and efficient intervention path at the end of the risk chain.

Emergency response path: F15 (community resources) → F17 (emergency mechanism) → F18 (rescue effectiveness). This path focuses on the disaster reduction phase after injury occurs, emphasizing the foundational role of pre-incident emergency preparedness. The model clearly outlines the transmission chain from community resource foundations to on-site response mechanisms, and then to final rescue effectiveness, echoing the continuously evolving concept of the “drowning survival chain,” emphasizing the continuity from on-site first aid to professional treatment as crucial (31). Among them, F17 (emergency mechanism) is the key hub connecting resources and outcomes. The World Health Organization (WHO) guidelines clearly state that community-level preparations (F15), such as establishing early warning systems, training the public in CPR, and ensuring unobstructed emergency access, are among the most effective strategies for reducing drowning deaths and sequelae (23). This collectively confirms the rationality of this path, that a strong community system is the cornerstone for building effective emergency mechanisms.

### ANP weight analysis: quantitative ranking of relative importance of factors

Based on the structural relationships revealed by the ISM model, the ANP model further quantified the global weights of each factor in the networked mutual influence, thereby determining the precise priorities for risk intervention.

High-weight factors (weight > 0.1): The sum of the weights of F4 (risky behavior, 0.2806) and F13 (caregiver behavior and supervision, 0.2326) exceeds 0.5, constituting the absolute core of the risk system. This result strongly confirms in quantitative terms that the focus of drowning risk prevention and control for preschool children must concentrate on the “child-caregiver” binary behavioral interaction. Research has confirmed that the lack of effective supervision is the primary proximate cause in the vast majority of drowning incidents involving young children, with its direct influence being the most prominent in the event chain (49, 50). F5 (lack of immediate response and protection, 0.1469) as another key behavioral node emphasizes the importance of response capability at the moment of the incident. Studies on the use of life jackets and other immediate protections also indicate that they play an irreplaceable role in determining whether injuries occur at critical moments (23, 29), supporting the rationality of the high weight assigned to it by ANP from an empirical perspective.

Medium-weight factors (weight 0.01–0.1): This range includes factors such as F3, F2, F14, F6, F18, F9, etc. They are mostly important risk moderators and barrier factors, such as water skills training (F3) and protective fences (F9), which systematic reviews have confirmed to have clear but not absolute protective effects, existing as important “buffer zones,” whose effectiveness often depends on the satisfaction of core conditions such as caregiver supervision (F13) (39). This explains their status as important supporting nodes in the ANP network rather than primary driving nodes.

Low/zero direct weight factors (weight ≤0.01): The ANP global weights of factors such as F1, F8, F15, F16 are 0. This does not mean they are unimportant, but reveals the differences in perspectives between ISM and ANP: ANP weights reflect the direct influence and activity of factors in the network, while ISM hierarchy reflects their structural importance and foundational nature. For example, in communities where socioeconomic disadvantages (F16) are widespread and severe, their impact is fundamental, but in network analyses targeting specific behavioral nodes, their causal pathways are completely mediated by more proximate factors (such as F13), thus exhibiting low direct weight. This finding suggests that decision-makers need to distinguish between “treating symptoms” (targeting high-weight direct factors) and “treating root causes” (targeting bottom-level root factors) strategies. Although interventions targeting high-weight behavioral factors (such as strengthening supervision) can achieve quick gains, if fundamental issues such as the lack of community support systems (F15) and family economic pressures (F16) are not addressed, the results of behavioral changes may be difficult to sustain. This emphasizes the necessity of combining the “intervention priorities” provided by ANP with the “system structure” revealed by ISM, implementing a dual approach of addressing both symptoms and root causes to achieve lasting prevention.

### Construction of an integrated prevention and control strategy system

Based on the integrated analysis of ISM and ANP, this study obtained a dual perspective on the “system structure” and “relative weights” of risk factors, making the conclusions both deep and precise. Following the principle of “structural layering, weight prioritization,” this study constructed a three-level interconnected systematic prevention and control strategy system.

For high-level, high-weight factors: Implement precise behavioral interventions. The core goal is to directly curb F4 (children’s risky behavior) and strengthen F13 (effective supervision by caregivers). Specific measures: (1) Develop safety education courses based on real scenarios and gamification, focusing on training children’s risk identification and safe behavior decision-making abilities. (2) Utilize digital media and community lectures to conduct high-intensity, situational caregiver training, emphasizing the principles of “active, uninterrupted, close-range” supervision, and provide special warnings for high-risk periods (such as summer afternoons). (3) Promote and mandate the wearing of qualified floating devices for children in family and public water areas to directly ensure immediate safety.

For intermediate factors: Strengthen physical and environmental barriers. The core goal is to weaken intermediate transmission factors and effectively cut off risk transmission paths. Specific measures: (1) Formulate and enforce standards for protective facilities in family and public water areas, popularize self-locking fences, and ensure that water storage containers are sealed to physically isolate hazards. (2) Clarify the responsibilities of various water area management entities, increase prominent warning signs and monitoring systems, and enhance on-site patrol forces during dangerous periods. (3) Implement mandatory regular safety inspections and maintenance for water entertainment facilities, boats, etc., to eliminate risks caused by equipment defects.

For bottom-level root factors: Strengthen the social ecological foundation. The core goal is to improve the macro environment that fosters risk from the system’s roots. Specific measures: (1) Promote the inclusion of systematic water safety education into preschool education and community public service systems, achieving the popularization and institutionalization of preventive knowledge. (2) For low-income families, provide home safety renovations, free life-saving equipment, or public custody services through government subsidies and social funding to reduce risk exposure disparities caused by economic conditions. (3) Integrate medical, firefighting, community, and other resources to improve drowning emergency plans and conduct regular drills, comprehensively enhancing emergency response mechanisms and rescue recovery effectiveness.

### Study limitations and future research

This study has several limitations. First, the data primarily reflects the perspectives of professionals in specific regions during the study period, which may not fully capture all local or temporally varying contexts. Second, the model, while systematic, is based on expert judgment and cross-sectional data, limiting dynamic causal inference. Future research should aim to validate this factor structure and hierarchy with longitudinal or incident-based data, and to adapt and test the proposed intervention strategies in diverse socio-cultural and environmental settings to assess their practical effectiveness.

## Conclusion

This study, based on the analysis of the ISM-ANP integrated model, indicates that the drowning risk for preschool children is a typical complex system process. Its transmission mechanism follows a clear path: originating from deep-rooted social economic and community environmental factors (roots), mediated by the key intermediaries of caregiver cognition and behavior (hubs), catalyzed by physical environmental defects (conditions), ultimately manifesting as children’s risky behavior and lack of immediate protection (direct manifestations). This systematic deconstruction reveals that risk is not triggered by isolated factors but is the result of dynamic interactions among multi-level factors. Correspondingly, an effective risk prevention and control system must be a multi-level, multi-strategy collaborative project. Based on the principle of “structural layering, weight prioritization”: in the short term, focus on the child-caregiver binary behavioral interaction, achieving rapid risk interruption of high-weight direct factors through precise behavioral interventions. In the medium term, aim to strengthen physical environments and supervisory barriers, cutting off key paths of risk transmission through engineering modifications and enhanced supervision, thereby improving the inherent resilience of the entire system. In the long term, focus on optimizing the social ecological foundation, fundamentally dissolving the soil that fosters risk through policy support, equitable resource distribution, and emergency network construction. Only by adopting this systematic governance plan of “treating both symptoms and root causes, layered strategies” can a collaborative and interconnected three-dimensional protection system be constructed, effectively safeguarding the life safety and healthy development of preschool children.

## Data Availability

The raw data supporting the conclusions of this article will be made available by the authors, without undue reservation.

## References

[1] World Health Organization. Global Report on Drowning: Preventing a Leading killer. Geneva: WHO (2014).

[2] Chinese Center for Disease Control and Prevention, National Center for Chronic and Non-communicable Disease Control and Prevention. Chinese Report on Childhood Injuries. Beijing: People's Medical Publishing House (2021).

[3] Shen J Pang S Schwebel DC. Cognitive developmental factors in unintentional drowning risk in preschool children. J Pediatr Psychol. (2016) 41:555–65. doi: 10.1093/jpepsy/jsv104, 26546476 PMC4888110

[4] Meddings D Hyder AA. The social and economic burden of drowning: a systematic review. Int J Environ Res Public Health. (2019) 16:343031527436

[5] Kovacevic P Dragic S Jandric M Momcicevic D Malesevic V Kovacevic T . Does adjunctive hemoadsorption provide benefit in the management of ischemia-reperfusion syndrome following near-drowning? A case report. Front. Med. (Lausanne). 11:1341156. doi: 10.3389/fmed.2024, 38633302 PMC11021721

[6] Hossain MJ Al-Mamun M Alam M Khatun MR Sarker MMR Islam MR. Child drownings in Bangladesh: need for action. BMJ Paediatr Open. (2022) 6:e001464. doi: 10.1136/bmjpo-2022-001464, 36053622 PMC9171250

[7] Tsarbou C Liveris NI Xergia SA Papageorgiou G Kvist J Tsepis E. ACL injury etiology in its context: a systems thinking, group model building approach. J Clin Med. (2024) 13:4928. doi: 10.3390/jcm13164928, 39201070 PMC11355078

[8] Chen X Qiao W. A hybrid STAMP-fuzzy DEMATEL-ISM approach for analyzing the factors influencing building collapse accidents in China. Sci Rep. (2023) 13:19745. doi: 10.1038/s41598-023-46778-6, 37957194 PMC10643550

[9] Sun Y Qu X Hu B Liu M Zhu X. Risk factors for disease aggravation in older patients with non-communicable diseases: interpretive structural and hierarchical holographic modelling. J Adv Nurs. (2025) 81:3771–85. doi: 10.1111/jan.16503, 39373056

[10] Gong J Li J Tan L Zhang L Wang Y Zhao J. Natural gas purification plants based on interpretive structural models and Bayesian networks. ACS Omega. (2025) 10:20026–37. doi: 10.1021/acsomega.5c02129, 40415862 PMC12096211

[11] Peng JL Liu X Peng C Shao Y. Comprehensive factor analysis and risk quantification study of fall from height accidents. Heliyon. (2023) 9:e22167. doi: 10.1016/j.heliyon.2023.e22167, 38107312 PMC10724537

[12] Aragonés-Beltrán P Pastor-Ferrando JP García-García F Pascual-Agulló A. An analytic network process approach for siting a municipal solid waste plant in the metropolitan area of Valencia (Spain). J Environ Manag. (2010) 91:1071–86. doi: 10.1016/j.jenvman.2009.12.007, 20080331

[13] Kamali MA Eghbalizarch M Jahanmahin R Masoud S. Defining the criteria for selecting the right extended reality systems in healthcare using fuzzy analytic network process. Sensors (Basel). (2025) 25:3133. doi: 10.3390/s25103133, 40431924 PMC12115641

[14] Tran LT Knight CG O'Neill RV Smith ER. Integrated environmental assessment of the mid-Atlantic region with analytical network process. Environ Monit Assess. (2004) 94:263–77. doi: 10.1023/B:EMAS.0000016893.77348.67, 15141460

[15] Molinos-Senante M Gómez T Caballero R Hernández-Sancho F Sala-Garrido R. Assessment of wastewater treatment alternatives for small communities: an analytic network process approach. Sci Total Environ. (2015) 532:676–87. doi: 10.1016/j.scitotenv.2015.06.059, 26119382

[16] Cummings P Koepsell TD Mueller BA. Methodological challenges in injury epidemiology and injury prevention research. Annu Rev Public Health. (1995) 16:381–400. doi: 10.1146/annurev.pu.16.050195.002121, 7639878

[17] Fritch WM Agnew J Rosman L Cadorette MA Barnett DJ. Application of the Haddon matrix to COVID-19 prevention and containment in nursing homes. J Am Geriatr Soc. (2021) 69:2708–15. doi: 10.1111/jgs.17358, 34235743 PMC8447078

[18] Hutton A Savage C Ranse J Finnell D Kub J. The use of Haddon's matrix to plan for injury and illness prevention at outdoor music festivals. Prehosp Disaster Med. (2015) 30:175–83. doi: 10.1017/S1049023X15000187, 25723292

[19] Willcox-Pidgeon SM Peden AE Scarr J. Exploring children's participation in commercial swimming lessons through the social determinants of health. Health Promot J Austr. (2021) 32:172–81. doi: 10.1002/hpja.335, 32187399

[20] Yusuf S Jones JL Camp EA McCallin TE. Drowning prevention counselling by paediatricians to educate caregivers on water safety. J Paediatr Child Health. (2022) 58:1584–93. doi: 10.1111/jpc.16049, 35665978

[21] Dimmer A Proulx KR Guadagno E Gagné M Perron PA Wissanji H. Beneath the surface: a retrospective analysis of pediatric drowning trends & risk factors in Quebec. J Pediatr Surg. (2025) 60:162184. doi: 10.1016/j.jpedsurg.2025.162184, 39893841

[22] Davoudi-Kiakalayeh A Barshan J Emami Sigaroudi F Mirak HM Naseri Alavi SA. The application of the Haddon matrix in identifying drowning prevention solutions in the north of Iran. Heliyon. (2023) 9:e16958. doi: 10.1016/j.heliyon.2023.e16958, 37484249 PMC10361018

[23] World Health Organization. Preventing Drowning: An Implementation Guide. Geneva: World Health Organization (2017).

[24] Chinese Center for Disease Control and Prevention, National Center for Chronic and Non-communicable Disease Control and Prevention. Child Drowning Prevention: Effective Supervision is Key. Beijing: People's Medical Publishing House (2019).

[25] Du Q. SPSS Statistical Analysis. Beijing: Posts and Telecom Press (2018). p. 275–7.

[26] Miao X Chen Y Mi C. Research on customer satisfaction of Tangshan Hot Springs based on ISM and online reviews. Chin J Manage Sci. (2019) 27:186–94.

[27] Reason J. Human error: models and management. BMJ (Clin Res Ed). (2000) 320:768–70. doi: 10.1136/bmj.320.7237.768, 10720363 PMC1117770

[28] Cohen N Scolnik D Rimon A Balla U Glatstein M. Childhood drowning: review of patients presenting to the emergency departments of 2 large tertiary care pediatric hospitals near and distant from the sea coast. Pediatr Emerg Care. (2020) 36:e258–62. doi: 10.1097/PEC.0000000000001394 29406474

[29] Moreland B Ortmann N Clemens T. Increased unintentional drowning deaths in 2020 by age, race/ethnicity, sex, and location, United States. J Saf Res. (2022) 82:463–8. doi: 10.1016/j.jsr.2022.06.012, 36031277 PMC9418042

[30] Claesson A Lindqvist J Herlitz J. Cardiac arrest due to drowning--changes over time and factors of importance for survival. Resuscitation. (2014) 85:644–8. doi: 10.1016/j.resuscitation.2014.02.006, 24560828

[31] Dezfulian C McCallin TE Bierens J Dunne CL Idris AH Kiragu A . 2024 American Heart Association and American Academy of Pediatrics focused update on special circumstances: resuscitation following drowning: an update to the American Heart Association guidelines for cardiopulmonary resuscitation and emergency cardiovascular care. Circulation. (2024) 150:e501–16. doi: 10.1161/CIR.0000000000001274, 39530204

[32] Gebru NM Goncalves PD Cruz RA Thompson WK Allegair N Potter A . Effects of parental mental health and family environment on impulsivity in preadolescents: a longitudinal ABCD study®. Front Behav Neurosci. (2023) 17:1213894. doi: 10.3389/fnbeh.2023.1213894, 37942273 PMC10628051

[33] Alschuler KN Whibley D Alberts NM Kaylor M Kratz AL. The physical and psychological experience of rowing the North Atlantic solo and unassisted. Wilderness Environ Med. (2020) 31:144–50. doi: 10.1016/j.wem.2019.12.008, 32229171

[34] Lukaszyk C Mittal S Gupta M das R Ivers R Jagnoor J. The impact and understanding of childhood drowning by a community in West Bengal, India, and the suggested preventive measures. Acta Paediatr. (2019) 108:731–9. doi: 10.1111/apa.14592, 30252948

[35] Lassen NM Steenhoff T Egmose I Cleal B Skovgaard Væver M. Experienced barriers and facilitators of change in a video-feedback intervention among parents of preschool children with externalizing behaviors: a qualitative study. Infant Ment Health J. (2026) 47:e70064. doi: 10.1002/imhj.70064 41486089

[36] Begum M Mamun MA Almerab MM Servidio R Soraci P Mamun F. Problematic gaming behavior among adolescents in Bangladesh. Psychol Rep. (2026) 16:3187. doi: 10.1177/00332941251413187, 41545007

[37] Al-Mamun M Alam M Hossain MJ Khatun MR Das PK Alam F . Child drowning and associated risk factors: findings from a qualitative study in Bangladesh. Health Sci Rep. (2023) 6:e1380. doi: 10.1002/hsr2.1380, 37396561 PMC10308348

[38] Agam A Godler Y Calif E. Child drowning mortality in Israel: trends and measures for prevention. J Saf Res. (2024) 89:224–33. doi: 10.1016/j.jsr.2024.02.002, 38858046

[39] Ashraf L Zia N Vincenten J Mackay JM Agrawal P Green A . Effectiveness of interventions to prevent drowning among children under age 20 years: a global scoping review. Front Public Health. (2024) 12:1467478. doi: 10.3389/fpubh.2024.1467478, 39811781 PMC11729736

[40] Scarr JP Jagnoor J. Conceptual definition for drowning prevention: a Delphi study. Inj Prev. (2024) 30:145–52. doi: 10.1136/ip-2023-045085, 37945328 PMC10958290

[41] Joanknecht L Argent AC van Dijk M van As AB. Childhood drowning in South Africa: local data should inform prevention strategies. Pediatr Surg Int. (2015) 31:123–30. doi: 10.1007/s00383-014-3637-0, 25403485

[42] Shimony-Kanat S Orr D Falk A. Social and economic factors associated with child unintentional injury mortality in high-income countries. Inj Prev. (2024) 30:194–9. doi: 10.1136/ip-2023-045016, 38050075

[43] Liu Z Kong F Yin L Wang A Xiong L Xie D . Epidemiological characteristics and influencing factors of fatal drowning in children under 5 years old in Hunan Province, China: case-control study. BMC Public Health. (2019) 19:955. doi: 10.1186/s12889-019-7241-z, 31315598 PMC6637556

[44] Martins CB Mello-Jorge MH. Circumstances and factors associated with accidental deaths among children, adolescents and young adults in Cuiabá. Brazil Sao Paulo Med J. (2013) 131:228–37. doi: 10.1590/1516-3180.2013.1314459, 24141293 PMC10871837

[45] Ohri-Vachaspati P DeLia D DeWeese RS Crespo NC Todd M Yedidia MJ. The relative contribution of layers of the social ecological model to childhood obesity. Public Health Nutr. (2015) 18:2055–66. doi: 10.1017/S1368980014002365, 25374257 PMC4775271

[46] Oftedal A Larsen L Helland MS. Economic hardship during the Covid-19 pandemic and trajectories of parent-child relationships: a prospective longitudinal study among Norwegian families. Fam Process. (2025) 64:e70031. doi: 10.1111/famp.70031, 40211581

[47] Reitman D Currier RO Stickle TR. A critical evaluation of the parenting stress index-short form (PSI-SF) in a head start population. J Clin Child Adolesc Psychol. (2002) 31:384–92. doi: 10.1207/S15374424JCCP3103_10, 12149976

[48] Manteiga-Urbón JL Martínez-Isasi S Fernández-Méndez F Otero-Agra M Sanz-Arribas I Barcala-Furelos M . Tourniquet application in time-critical aquatic emergencies on a moving rescue water craft (RWC): can speed and precision coexist? Am J Emerg Med. (2024) 82:161–5. doi: 10.1016/j.ajem.2024.06.011, 38909551

[49] Petrass LA Blitvich JD Finch CF. Lack of caregiver supervision: a contributing factor in Australian unintentional child drowning deaths, 2000-2009. Med J Aust. (2011) 194:228–31. doi: 10.5694/j.1326-5377.2011.tb02950.x, 21381993

[50] McLellan-Lamarche S Keays G Foley BC Beaudoin C Friedman D Gagnon I . Mechanisms and products implicated in mild traumatic brain injury among children aged 0-5 years old presenting to Canadian emergency departments. Inj Prev. (2026):ip-2025-045929. doi: 10.1136/ip-2025-045929, 41513441

